# Generative AI in oncological imaging: Revolutionizing cancer detection and diagnosis

**DOI:** 10.18632/oncotarget.28640

**Published:** 2024-09-04

**Authors:** Yashbir Singh, Quincy A. Hathaway, Bradley J. Erickson

**Keywords:** generative AI, oncological imaging, personalized cancer screening

## Abstract

Generative AI is revolutionizing oncological imaging, enhancing cancer detection and diagnosis. This editorial explores its impact on expanding datasets, improving image quality, and enabling predictive oncology. We discuss ethical considerations and introduce a unique perspective on personalized cancer screening using AI-generated digital twins. This approach could optimize screening protocols, improve early detection, and tailor treatment plans. While challenges remain, generative AI in oncological imaging offers unprecedented opportunities to advance cancer care and improve patient outcomes.

The landscape of oncological imaging is undergoing a seismic shift, propelled by the rapid advances in generative artificial intelligence (AI). This transformative technology is not just enhancing our ability to detect and diagnose cancer; it’s redefining the entire paradigm of oncological care. As we stand on the cusp of this revolution, it’s crucial to examine the far-reaching implications of generative AI in cancer imaging and explore its potential to reshape the future of oncology. Generative AI methods are well-known for language, thanks to ChatGPT, and its many competitors. But generative AI techniques, e.g., generative adversarial networks (GANs) and denoising diffusion probability models(DDPMs), have also demonstrated remarkable capabilities in medical imaging [1, 2]. These models can generate synthetic medical images, enhance image quality, and even predict future progression of tumors. In oncological imaging, this translates to earlier detection, more accurate diagnosis, and improved treatment planning. One of the most promising applications of generative AI is in addressing the perennial challenge of data scarcity in medical imaging. Cancer, especially in its early stages, often presents subtle abnormalities that can be easily missed. By generating synthetic images of rare cancer types or early-stage tumors, AI can significantly expand the dataset available for training diagnostic algorithms.

Moreover, generative AI is proving invaluable in image enhancement. It can denoise low-dose CT scans, improve the spatial resolution of MRI images, and even reconstruct missing data in partial scans [1]. This not only improves diagnostic accuracy but also reduces the need for repeated scans, minimizes radiation exposure and reduces healthcare costs. But perhaps the most exciting frontier is the use of generative AI in predictive oncology. By analyzing current imaging data and generating potential future scenarios, these models can predict likely tumor growth patterns and treatment responses.

However, as we embrace this technological revolution, we must also grapple with its ethical implications [3]. The use of these generated (synthetic) images raises questions about patient privacy and data ownership. How do we ensure that the synthetic images generated by AI do not inadvertently reveal sensitive patient information? Furthermore, as AI becomes more integral to diagnosis, we must carefully consider issues of accountability and transparency. Who is responsible when an AI-assisted diagnosis proves incorrect? Despite these challenges, the potential benefits of generative AI in oncological imaging are too significant to ignore. As we continue to refine these technologies, we must strive for a balanced approach that maximizes their potential while addressing ethical concerns.

Now, let’s explore a unique angle that could further revolutionize cancer care: personalized cancer screening powered by generative AI. Imagine a future where each individual has a digital twin of their body, continuously updated with their latest health data [4]. Generative AI could use this personalized model to simulate potential cancer scenarios based on the individual’s unique genetic makeup, lifestyle factors, and environmental exposures. This would allow for truly personalized cancer screening protocols, moving beyond the current one-size-fits-all approach [5]. For instance, instead of recommending mammograms at a certain age for all women, an AI system could analyze an individual’s digital twin and determine the optimal screening schedule and modality. For someone with a higher risk of aggressive breast cancer, the AI might recommend more frequent screenings or additional imaging techniques. Conversely, for individuals at lower risk, it might suggest less frequent screenings, reducing unnecessary procedures and anxiety. This personalized approach could extend beyond screening to diagnosis and treatment planning. When a suspicious lesion is detected, generative AI could simulate its potential growth patterns based on the individual’s unique characteristics. This would allow oncologists to tailor treatment plans with unprecedented precision, potentially improving outcomes and reducing side effects. Moreover, this personalized model could be used to generate synthetic images of what potential cancers might look like in that specific individual. These images could be used to train diagnostic AI systems, creating a positive feedback loop that continually improves the accuracy of cancer detection for that person. The implications of such a system are profound. It could dramatically reduce overdiagnosis and overtreatment, a significant problem in cancer care. It could also lead to earlier detection of aggressive cancers in high-risk individuals, potentially saving lives. Furthermore, by optimizing screening protocols, it could make cancer screening more cost-effective, potentially allowing for broader implementation in resource-limited settings. Of course, realizing this vision of personalized cancer screening and diagnosis powered by generative AI faces significant challenges. It requires vast amounts of data, robust privacy protections, and careful validation to ensure safety and efficacy. However, as our understanding of cancer biology and our AI capabilities continue to advance; this future may be closer than we think.

In conclusion, generative AI is poised to revolutionize oncological imaging, offering new hope in our fight against cancer. As we navigate this exciting frontier, we must remain committed to ethical innovation, always keeping the patient at the center of our efforts [6, 7]. The future of cancer care is not just about better technology; it’s about smarter, more personalized care that gives each individual the best chance at a healthy life.
